# The prospective relationship between anxiety symptoms and eating disorder symptoms among adolescents: a systematic review and meta-analysis of a bi-directional relationship

**DOI:** 10.1007/s00787-024-02601-9

**Published:** 2024-11-07

**Authors:** Nora Trompeter, Ștefana Dârvariu, Anna V. Brieva-Toloza, Marie-Christine Opitz, Francisco Diego Rabelo-da-Ponte, Helen Sharpe, Sylvane Desrivieres, Ulrike Schmidt, Nadia Micali

**Affiliations:** 1 Great Ormond Street Institute of Child Health, UCL, 30 Guilford Street, London, WC1N 1EH UK; 2 Center for Eating and Feeding Disorders Research, Mental Health Center Ballerup, Copenhagen University Hospital, Mental Health Services CPH, Copenhagen, Denmark; 3 Department of Clinical Psychology, School of Health in Social Sciences, University of Edinburgh, Edinburgh, UK; 4 Social Genetic and Developmental Psychiatry Centre, Institute of Psychiatry, Psychology and Neuroscience, King’s College London, London, UK; 5 Department of Psychological Medicine, Institute of Psychiatry, Psychology and Neuroscience, King’s College London, London, UK

**Keywords:** Eating disorders, Disordered eating, Anxiety, Social anxiety, Longitudinal

## Abstract

Anxiety symptoms and eating disorder symptoms commonly co-occur in adolescents. However, there is divergent evidence on the prospective relationship between the two factors. This systematic review and meta-analysis summarises the existing literature on the prospective and bi-directional relationship between anxiety symptoms and eating disorder symptoms in adolescence. A systematic search across six databases was conducted on the 11th June 2024. Studies were included if they assessed the prospective relationship between anxiety symptoms and eating disorder symptoms, or vice versa, in adolescence. 19,591 studies were screened, of which 54 studies met inclusion criteria and were included in the full review. Four meta-analyses were conducted. Anxiety symptoms were associated with subsequent eating disorder symptoms, increases in eating disorder symptoms, and higher odds of eating disorders, including their onset. Conversely, eating disorder symptoms were associated with subsequent anxiety symptoms, increases in anxiety symptoms, and higher odds of subsequent anxiety disorders. Current evidence suggests that anxiety symptoms and eating disorder symptoms do not merely co-occur during adolescence, but are prospectively and bi-directionally linked. Further research is needed to understand the underlying mechanisms of this relationship, as well as individual differences in symptom trajectories.

**Supplementary Information:**

The online version contains supplementary material available at 10.1007/s00787-024-02601-9.

Anxiety symptoms and eating disorder symptoms are frequently experienced by adolescents [1–3]. Both are linked with heightened distress and increased risk of developing clinical full-blown disorders (i.e., anxiety disorders and eating disorders) and are highly likely to co-occur [4, 5]. Indeed, young women who experience eating disorder symptoms are 3–4 times more likely to also experience clinical levels of anxiety symptoms [6]. Despite this common co-occurrence, it is not clear whether symptoms share a common aetiology or have a causal link with one another [7]. Identifying the temporal relationship of symptoms, especially during adolescence, is critical for informing early intervention and prevention efforts that could target anxiety or eating disorder symptoms to prevent the onset of both anxiety disorders and eating disorders [8]. The current systematic review aimed to examine the prospective evidence linking anxiety symptoms and eating disorder symptoms to establish whether: a) anxiety symptoms temporally predict eating disorder symptoms, and/or b) eating disorder symptoms temporally predict anxiety symptoms.

## Anxiety symptoms and subsequent eating disorder symptoms

Most research to date has explored whether anxiety disorders predict the future onset of eating disorders [9–11], based on converging evidence within clinical samples that anxiety disorders tend to occur before eating disorder onset [4, 12]. This research suggests that the presence of any anxiety disorder, rather than a specific type of anxiety, is predictive of an increased risk for eating disorder development. While the examination of the anxiety and eating comorbidity at a diagnostic level has highlighted the co-occurrence of disorders, this approach lacks nuanced information on symptomatic associations that may further our understanding of the underlying aetiology of this comorbidity. More recent models of comorbidity in mental illness suggest that symptoms of multiple disorders could be explained by latent liability that influences the symptoms presentation of those disorders (e.g., behavioural symptoms), as well as their progression over time [13]. As such, it is pertinent to extend this line of research by investigating the comorbidity between anxiety disorders and eating disorders at the symptom level as well, to gain a deeper understanding of this comorbidity.

Additionally, many studies have relied on clinical samples and focused on adults retrospectively reporting the time-ordering of their disorder onset [5]. Such approaches may be biased due to recall and selection bias, questioning whether findings from adult clinical samples apply to adolescents experiencing sub-clinical symptoms of anxiety or eating pathology. Among adolescents, research suggests that the temporal relationship between anxiety symptoms and eating disorder symptoms may be more complicated than suggested by adult clinical studies. For example, research has found links between anxiety symptoms and eating disorder symptoms for some age groups (e.g., early adolescence and late adolescence/emerging adults), but not others (e.g., mid-adolescence) [14]. Indeed, a systematic review on eating disorder symptom trajectories in adolescents suggests that anxiety symptoms during middle childhood/early adolescence are associated with later onset of eating disorders [11]. Additionally, some anxiety symptoms, like worry and anxiety sensitivity, appear to be more predictive of future eating disorder symptoms compared to others [14]. Thus, a comprehensive overview of the existing literature is needed to gain a more complete understanding of how anxiety symptoms and eating disorder symptoms relate to one another over time.

## Eating disorder symptoms and subsequent anxiety symptoms

In contrast to cumulative research on investigating anxiety as a predictor for eating disorders, relatively few studies have examined whether eating disorder symptoms predict anxiety symptoms. Indeed, in previous reviews on the clinical comorbidity of anxiety disorders and eating disorders, only one study was identified that examined the prevalence of eating disorders within individuals with an anxiety disorder [15]. Within clinical populations, research suggests that adults with an anxiety disorder have higher odds of also meeting criteria for an eating disorder compared to adults without an anxiety disorder [16]. Further, eating disorders in adolescents have been linked with anxiety disorders in adulthood in the general population [17]. Emerging research is pointing to a bi-directional relationship, whereby anxiety disorders during adolescence predict eating disorders in adulthood and vice versa [18]. However, different patterns emerge for different disorders. Thus, a clearer understanding of the symptomatic associations is needed.

## Current review

In sum, current research indicates that (a) anxiety disorders and eating disorders are highly comorbid, (b) both anxiety symptoms and eating disorder symptoms are commonly experienced by adolescents, and (c) anxiety symptoms and eating disorder symptoms are positively associated.

Given these findings, this review aimed to provide a comprehensive and objective synthesis of the current literature on how anxiety symptoms and eating disorder symptoms relate to one another over time in adolescents. Such an overview may elucidate whether focusing on both anxiety symptoms and eating disorder symptoms as focal points for psychosocial interventions and preventive strategies is supported by evidence. In particular, the current review focused on adolescence as a key developmental period for the onset of eating disorder symptoms and heightened prevalence of anxiety symptoms [19].

## Methods

### Study protocol

The PRISMA guidelines for reporting systematic reviews and meta-analyses were followed [20]. The PRISMA checklist is listed in Supplementary Material 1. The review protocol was pre-registered with the PROSPERO database of systematic review protocols (#CRD42023425811) and on the open-science framework (https://doi.org/10.17605/OSF.IO/CE8D3). All deviations from the protocol are clearly outlined below.

## Study selection and literature search

Eligible studies were required to: (1) have quantitative data on at least two timepoints, (2) be conducted among adolescents from the general population (mean age 12–21 years), and (3) examine the prospective associations between anxiety and eating disorder symptoms. We considered studies that examined both general levels of anxiety or eating disorders, symptoms of specific disorders (e.g., anorexia nervosa, generalised anxiety disorder), and specific symptoms (e.g., binge eating, rumination). Studies were excluded if they: (1) included an intervention or clinical trial, (2) included cross-sectional data only, (3) did not include measures on anxiety or eating disorder symptoms, or (4) did not test the association between anxiety and eating disorder symptoms prospectively. Further decisions were made to exclude studies that included concepts relating to both anxiety and eating disorder symptoms (e.g., social appearance anxiety) in the absence of other anxiety or eating disorder measures. Studies in languages other than English were assessed by members of the research team with suitable language expertise, where possible. Remaining studies were excluded based on language as translation was deemed inappropriate. References for these studies are provided in Supplement 1.

Search terms listed below were combined using the Boolean AND operator:

i) Anxiety terms: “anxi*” OR “fear” OR “ruminat*” OR “worry*” OR “PTSD” OR “OCD” OR “generalized anxiety” OR “phobi*” OR “obsessi*” OR “compulsi*” OR “intrusive th*” OR “shyness” OR “nervousness”
ii) Eating disorder terms: “eating habits” OR “disordered eating” OR “eating disorder*” OR “eating pathology” OR “body image” OR “body dissatisfaction” OR “shape concerns” OR “weight concerns” OR “excessive exercise” OR “diet*” OR “restrictive eat*” OR “dietary restraint” OR “fast*” OR “intention to lose weight” OR “bulimi*” OR “binge*” OR “overeat*” OR “night eat*” OR “purg*” OR “laxative” OR “diuretic” OR “vomit*” OR “loss of control eat*” OR “emotional eat*”
iii) Adolescence terms: “teen*” OR “youth*” OR “adolescen*” OR “juvenile*” OR “young adult*” OR “young person*” OR “young people*” OR “young m#n” OR “young wom#n” OR “high school*”

The initial search on the databases was conducted on 12th May 2023 and updated on 11th June 2024. The search did not include a set start date, thus articles from the databases' inception were included. The search was conducted in MEDLINE, PsycINFO, Scopus, Web of Science, Embase, and ProQuest. We had originally planned to also include a systematic search in PsyArXiv to identify further grey literature. However, due to issues with using the exact search terms importing references into Covidence, and identifying duplicates based on peer-reviewed studies, a manual search was conducted on PsyArXiv to identify further relevant papers. The search and study selection was conducted by one author (NT), two authors (SD & ABT) screened 20% of studies. Screening was conducted independently, and conflicts were resolved among the authors. To avoid conflicts of interest, authors did not screen any published papers on which they were listed as authors.

## Assessment of study quality

The NIH Quality Assessment Tool for Observational Cohort and Cross-Sectional Studies checklist [21] was used to assess study quality of all included studies. Quality assessments were carried out independently and blindly by two authors (NT and MO). Any discrepancies were resolved through discussion between the two authors. To avoid conflicts of interest, authors did not rate any published papers on which they were listed as authors.

## Data extraction

All data extraction and coding was performed by the first author (NT) and two independent authors (SD & ABT) who extracted 100% of data for quality control. The following study information variables were extracted: author/s, date of publication, title of publication, publication type, number of participants, sample description (e.g., age, gender, ethnicity), length of follow-up, type of anxiety measure, type of eating disorder measure, association between anxiety and eating disorder symptoms and effect size(s).

## *Meta*-analysis

In addition to the planned narrative synthesis of all reviewed studies, four random effects meta-analyses were conducted as more papers than expected were identified in the systematic review. These meta-analyses were not part of the pre-registration and should be noted as a change from the original plan. Studies were included in the meta-analysis if they included broad anxiety measures (e.g., overall symptoms) rather than specific symptoms (e.g., rumination) to allow for meaningful comparisons. Studies reporting on generalised anxiety or social anxiety were included, whereas studies reporting on obsessive compulsive disorder (OCD), post-traumatic stress disorder (PTSD), or panic symptoms were excluded from the meta-analysis due to symptom-specificity, but are discussed in the narrative synthesis. Studies reporting on either broad eating disorder symptoms (e.g., overall symptoms) or specific behaviours (e.g., dietary restraint) were included in the meta-analysis.

To account for differences in measurement types, we conducted four different meta-analyses: 1. anxiety symptoms predicting eating disorder symptoms (dichotomous), 2. anxiety symptoms predicting eating disorder symptoms (continuous), 3. eating disorder symptoms predicting anxiety symptoms (dichotomous), 4. eating disorder symptoms predicting anxiety symptoms (continuous).

Random effects models were used, due to the variation across study characteristics meaning that no single effect size could be assumed. Effect sizes (Odds ratios/risk ratios or Pearson’s *r*) were directly obtained from studies or calculated based on frequencies in the case of odds ratios. For studies reporting continuous measures, the pooled effect size included un-adjusted bi-variate correlations only (i.e., Pearson’s *r*). For studies reporting dichotomous measures, the pooled effect size included both adjusted and un-adjusted estimates (i.e., odds ratios/risk ratios).

Studies not reporting sufficient information to obtain effect sizes were not included in the meta-analysis (e.g., reporting results of regression models only). If studies reported multiple effect sizes, we chose to retain effect sizes with the longest delay between measurements. If effect sizes were reported for separate outcomes or by gender, multiple effect sizes per study were included. Sensitivity analyses using a multi-level meta-analysis clustering by study were conducted to account for study dependence.

Statistical analysis was performed with R version 3.6.0, using the *metafor* package. Heterogeneity was examined using the *I*^2^ and *Cochran’s Q* statistics [22]. In general, *I*^2^ values of 25% reflect low heterogeneity, 50% moderate heterogeneity and > 75% high heterogeneity [22]. *Cochran’s Q* is the weighted sum of squared differences between observed effects and weighted average effect, whereby a significant *Cochran’s Q* statistic indicates heterogeneity. In case of high heterogeneity, we examined the moderating effects of age at baseline and length of follow-up using meta-regressions treating both constructs as a continuous variable. Other potential moderators that were considered, but ultimately not included due to high degrees of heterogeneity in measures or reporting included eating disorder type, different anxiety symptoms and gender. Small study bias was examined using funnel plots and Egger’s regression test (Egger et al., 1997). Study outliers were tested to determine potentially influential studies. Overlap of included studies within each meta-analysis is shown in Supplementary Fig. 1.

## Results

### Study selection

**Fig. 1:**
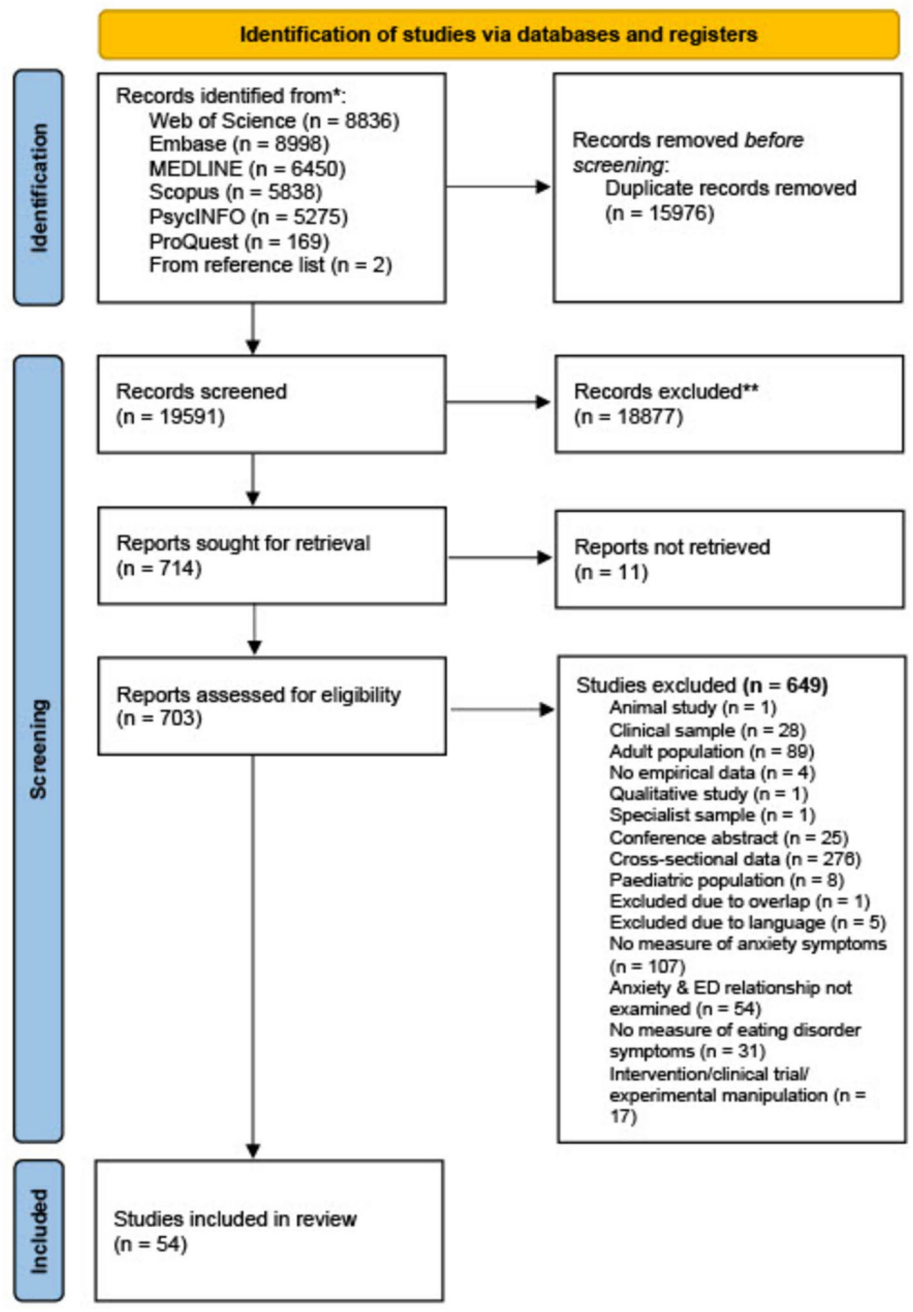
PRISMA flowchart

The searches of the online databases generated 35,568 records. Following de-duplication, the abstracts and titles of 19,591 records were screened. 714 full-text studies were screened. Two additional studies were identified as suitable through PsyArXiv or references of studies which met the eligibility criteria. See Fig. 1 for PRISMA flowchart. Agreement between raters was acceptable in both the title/abstract screening stage (86.2–88.7% agreement; Cohen’s kappa: 0.35–0.44), and full-text screening stage (78.8–93.2% agreement; Cohen’s kappa: 0.27–0.63).

Several eligible studies used the same study sample and were assessed for overlap. LeGrange et al. [23] and Linardon et al. [24] both used data from the Australian Temperament Project. LeGrange et al. [23] reported on early anxiety predicting adolescent eating disorder symptoms, whereas Linardon et al. [24]reported on adolescent eating disorder symptoms predicting adult anxiety. As both studies used different waves of participant data and examined different relationships, both studies were retained. Lloyd et al. [25], Micali et al. [26], Micali et al. [17], and Schaumberg et al. [14] all used data from the Avon Longitudinal Study of Parents and Children (ALSPAC). Lloyd et al. [25], used anxiety diagnosis data at ages 13/14 and 15/16 to predict fasting at ages 15/16 & 17/18. Micali et al. [26] examined latent classes of eating disorder symptoms at ages 14 and 16 to predict anxiety diagnosis at ages 16 and 18. Micali et al. [17] examined eating disorder diagnoses at age 14 and how they predict anxiety disorders at age 16. Lastly, Schaumberg et al. [14] used anxiety symptom clusters at age 10 to predict disordered eating at age 14. Given the overlap of waves and measures between the two Micali et al. studies, we chose to retain only Micali et al. [17] for the purpose of the review, as it provided more detailed information on different eating disorder symptoms. We further chose to retain Lloyd et al. [25] and Schaumberg et al. [14] due to different measures and waves of data collection used. Lastly, Levinson and Rodebaugh [27], Levinson et al. [28], & Sala and Levinson [29] all used data from the same US undergraduate sample. As the sample and outcome measures were identical for all three studies, we decided to merge study findings into one single study subsequently referred to as Levinson & Sala, et al. (2016). After this consolidation, 54 studies were included in the review.

## Study characteristics and quality assessment

Table 1 shows the study characteristics of the included studies. Most studies were conducted in Western countries in mixed-gender samples. Studies covered a range of age groups and varied in delays between measurement points. In terms of study quality, all included studies were rated as either ‘good’ or ‘fair’ and no studies were excluded from the review based on study quality. Full information on quality ratings can be found in Table 2. Overall, methodological gaps were identified in the reporting of power analyses and participant retention over time.

**Table 1:** Study characteristics

First authors, Year	Country	Length of follow-up	N	Gender (% girls)	Mean age (M), standard deviation (SD)	Race/Ethnicity	Anxiety measure	Eating pathology measure
Abebe et al. [30]	Norway	6 years	3150	54.9%	Males: 16.3 (1.7); Females: 16.5 (1.7)	NR	6 items derived from the Hopkins Symptom Checklist	Bulimic Investigatory Test (BITE)
Allen et al. [31]	Australia	3 years	1383	51%	14.0 (0.2)	NR	The 21-item Depression Anxiety Stress Scale (DASS)	Child Eating Disorder Examination (ChEDE)
Balantekin et al. [32]	US	8 years	158	100%	7.0 (NR)	White	28-item Anxiety subscale on the Children's Manifest Anxiety Scale	Binge Eating Scale (BES
Bardone-Cone et al. [33]	US	14 months	237	100%	18.7 (1.0)	69.1% non-Hispanic Caucasian/White, 7.6% African American/Black, 11.4% Hispanic/Latina, 5.5% Asian, and 6.4% multiple race/ethnicities	Spielberger State-Trait Anxiety Inventory (STAI)	Eating Attitudes Test-26 (EAT-26)
Benjet et al. [34]	Mexico	8 years	1071	57%	12–17 (range)	NR	WHO Composite International Diagnostic Interview 3.0 (CIDI)	WHO Composite International Diagnostic Interview 3.0 (CIDI)
Bodell et al. [35]	US	8 weeks	270	100%	18.7 (1.42)	27% White, 43% Black, 25% Latina, 1% Asian, and 4% biracial	Beck Anxiety Inventory (BAI)	Eating Disorder Inventory (EDI)
Buckner et al. [18]	US	14 years	816	53.7%	16.6 (1.2)	Primarily Caucasian (59%)	Schedule for Affective Disorders and Schizophrenia for School-Age Children (K-SADS) at T1; Longitudinal Interval Follow-up Evaluation and the Structured Clinical Interview for DSM-IV, non-patient version (SCID-I/NP) at T4	Schedule for Affective Disorders and Schizophrenia for School-Age Children (K-SADS) at T1; Longitudinal Interval Follow-up Evaluation and the Structured Clinical Interview for DSM-IV, non-patient version (SCID-I/NP) at T4
Bufferd et al. [36]	US	12 years	609	45.5%	3.0 (NR)	9.9% Hispanic or Latino, 90.1% not Hispanic or Latino	Child Behavior Questionnaire (CBQ) & Preschool Age Psychiatric Assessment (PAPA)	Body dissatisfaction subscale from the Minnesota Eating Behavior Survey (MEBS) & Kiddie Schedule for Affective Disorders and Schizophrenia Present and Lifetime Version (K- SADS-PL)
Cronce et al. [37]	US	Baseline, 12-month, and 24-month follow-up	425	100%	20.9 (2.1)	72.9% White, 8.0% Black/African American, 2.1% Asian/Asian American, 0.2% American Indian/Alaskan native, and 13.9% multiracial	PTSD Checklist-Specific version (PCL-S)	Single-item binge eating
Dworschak et al. [38]^1^	US	21-day daily diary, plus one year follow-up	139	52.3%	11.9 (2.1)	69.8% White, 7.2% Black, 7.2% Asian, 6.5% Latino, 3.6% Arab, 1.4% American Indian, 4.3% Mixed	Children's Response Style Questionnaire	Eating Disorder Examination- Questionnaire (EDE-Q)
Fairweather-Schmidt and Wade [39]	Australia	1.15 years (SD = 0.17)	669	100%	NR	Predominantly white	Child Anxiety Sensitivity Index (CASI) & Just Right from the Vancouver Obsessive Compulsive Inventory	EDE interview and Perceived Pressure to be Thin
Fitzsimmons-Craft et al. [40]	US	5 months	276	100%	African-American: 19.0 (1.6); White: 18.6 (1.1)	65% Caucasian non-hispanic/White, 35% African American/Black	Spielberger State-Trait Anxiety Inventory (STAI)	Weight Concern and Shape Concern subscales of the Eating Disorder Examination-Questionnaire (EDE-Q)
Fitzsimmons-Craft et al. [40]	US	10 weeks	406	100%	18.6 (1.0)	92.4% Caucasian	Spielberger State-Trait Anxiety Inventory (STAI)	Cognitive Restraint subscale of the Three Factor Eating Questionnaire (TFEQ-R) & Bulimia subscale of the Eating Disorder Inventory (EDI)
Gilbert and Meyer [41]	UK	33 weeks	143	100%	18.7 (1.7)	NR	Fear of Negative Evaluation Scale (FNE) & Hospital Anxiety and Depression Scale (HADS)	Eating Disorders Inventory (EDI)
Goodwin et al. [42]	UK	2 years	369	59.9%	12.9 (0.7)	White British (93.2%)	Hospital Anxiety and Depression Scale (HADS) & Spence Child Anxiety Scale, Obsessive Compulsive Subscale (SCAS-OC)	Compulsive Exercise Test (CET)
Hamann et al. [43]	US	5 months	119	100%	19.1 (1.4)	81.5% Caucasian, 10.1% Other, 6.7% African American, and 1.7% Hispanic	Brief Fear of Negative Evaluation Scale (Brief-FNE)	The Bulimia Test ‚Revised (BULIT-R)
Hanback [44]	US	15 months	1263	57%	15.6 (0.6)	56.5% White, not of Hispanic origin, 17.2% Hispanic, 16.9% Black, not of Hispanic origin, 5.3% other or unknown, 0.2% American Indian or Alaskan Native, 4% Asian or Pacific Islander	Mood and Anxiety Symptom Questionnaire	Youth risk behavior survey (YRBS)
Hautala et al. [45]	Finland	1 year	372	57%	15–16 (range)	NR	Single item from Beck Depression Inventory	Sick, Control, One stone, Fat, Food (SCOFF)
Herpertz-Dahlmann et al. [46]	Germany	6 years	771	54.5%	14.3 (2.0)	NR	Screen for Child Anxiety-Related Emotional Disorders questionnaire (SCARED)	Sick, Control, One stone, Fat, Food (SCOFF)
Holm-Denoma and Hankin [47]	US	5 weeks between each time point	350	57%	14.5 (1.4)	53% White, 21% African American, 13% Latino, 7% biracial or multiracial, and 6% Asian or Pacific Islander	Children’s Response Style Questionnaire (CRSQ)—rumination subscale	Eating Disorder Diagnostic Scale (EDDS)
Hou et al. [48]	China	3 years	471	100%	18.6 (0.8)	NR	The anxiety symptoms subscale in SCL-90	EDI—bulimia subscale
Isaksson et al. [49]	US	1 year	2612	53.5%	12.8 (1.3)	60.6% African American, 25.7% Hispanic American, 13.7% Caucasian	Child Post-Traumatic Stress—Reaction Index	Eating Problem Scale (EPS)
Johnson et al. [50]	US	2.5 years and 8 years from baseline	717	51%	13.8 (2.6)	91% White	Parent and youth versions of the Diagnostic Interview Schedule for Children (DISC-I)	Parent and youth versions of the Diagnostic Interview Schedule for Children (DISC-I)
Kidwell et al. [51]		7 years	170	51.7%		64.9% European American, 18.4% multiracial, 14.4% Hispanic/Latino, 1.7% African American, and 1.0% Asian American	The Revised Child Manifest Anxiety Scale- Short Form Second Edition (RCMAS-2)	Dutch Eating Behaviors Questionnaire
Lacroix et al. [52]	US	At ages 11, 14, 18, 21, 25, and 29 years	760	100%	11	>95% White	State-Trait Anxiety Measure for Children	Minnesota Eating Behavior Survey
Le Grange et al. [23]	Australia	15 years	1300	51.3%	NR	NR	Single-item questions	Eating Disorder Inventory (EDI)
Lee and Vaillancourt [53]	Canada	Yearly for 4 years	657	53%	10.9 (0.4)	71% White	Behavior Assessment System for Children-2 (BASC-2)	Short Screen for Eating Disorders
Levinson and Sala, (2016) [29]	US	6 months	300	100%	18 (median)	Caucasian (60.7%), Asian (19.3%), Black (4.0%), Hispanic (2.7%), multi-racial (5.0%))	The Social Interaction Anxiety Scale (SIAS) & Brief Fear of Negative Evaluation (BFNE) scale & Penn State Worry Questionnaire (PSWQ)	The Eating Disorder Inventory-2 (EDI-2)
Lieb et al. [54]	Germany	10 years	2210	NR	18.3 (3.3)	NR	Munich-Composite International Diagnostic Interview (DIA-X/M-CIDI)	Munich-Composite International Diagnostic Interview (DIA-X/M-CIDI)
Lim et al. [55]	UK	2 years, yearly assessments	324	67%	13.4 (0.7)	74.69% Caucasian	Revised Children Anxiety and Depression Scale (RCADS)	Three-Factor Eating Questionnaire-R18 (TFEQ-R18)
Linardon et al. [24]	Australia	12 years	1568	51.3%	15–16 (range)	NR	Depression Anxiety Stress Scales (DASS)	Eating Disorder Inventory (EDI)
Lloyd et al. [25]	UK	4 years	2406	100%	13 years, 10 months (median)	white (87.6%), other (1.5%), missing (10.8%)	Development and Wellbeing Assessment (DAWBA)	McKnight Risk Factor survey
Loose et al. [56]	Canada	11 years	1316	57.7%	12 (NR)	NR	Generalized Anxiety Disorder (GAD-7) scale	Sick, Control, One stone, Fat, Food (SCOFF)
Magson et al. [57]	Australia	3 years, yearly assessments	528	49.9%	11.2 (0.6)	White (81.9%), Asian (6.4%), Middle Eastern (1.5%), or other (10.2%; Eurasian 4.4%, European 2.1%, The Americas 1.3%, Indian 1.1%, Maori/Islander 0.9%, unknown 0.4%)	Social anxiety subscale of the Spence Children’s Anxiety Scale (SCAS-C)	Children’s Eating Attitude Test (chEAT)
McLaughlin et al. [58]	US	7 months	1065	48.8%	NR	13.2% White, 11.8% Black, 56.9% Hispanic/Latino, 2.2% Asian/Pacific Islander, 0.2% Native American, 0.8% Middle Eastern, 9.3% Biracial/Multiracial, and 4.2%other racial/ethnic groups	Children’s Response Styles Questionnaire (CRSQ)—rumination	Children’s Eating Attitudes Test (ChEAT)
Micali et al. [17]	UK	2 years	6140	55.5%	14.0 (0.2)	87.2% Caucasian	Development and Wellbeing Assessment (DAWBA)	Youth Risk Behavior Surveillance System questionnaire, McKnight Risk Factor Survey, & Development and Wellbeing Assessment (DAWBA)
Minnich et al. [59]	US	8 weeks	302	0%	19.2 (1.3)	88.8% White, 1.3% African-American or Black, 5.3% Asian, 2% Hispanic/Latino, and 2.7% Other	Beck Anxiety Inventory (BAI)	Eating Disorder Inventory (EDI), Binge Eating Scale (BE), Drive for Muscularity Scale (DMS)
Nolen-Hoeksema et al. [60]	US	4 years, yearly assessment	496	100%	13.5 (0.7)	2% Asian or Pacific Islanders, 7% African Americans, 68% Caucasians, 18% Hispanics, 1% Native Americans, and 4% other or mixed racial heritage	Rumination Scale of the Response Styles Questionnaire	Eating Disorder Examination (EDE)
Parker et al. [61]	US	EMA study over 14 days	100	45%	12.8 (2.7)	47% non-Hispanic White, 19% non-Hispanic Asian American, 16% non-Hispanic Black, 11% non-Hispanic mixed race, 7% Hispanic	Brunel Mood Scale	Eating Disorder Examination (EDE)
Patton et al. [62]	Australia	10 years	1943	NR	14.5 (NR)	NR	Revised Clinical Interview Schedule (CIS-R)	Branched Eating Disorders Test (BET)
Puccio et al. [63]	Australia	2 years	189	NR	15.0 (0.4)	Parents from Australia (74.1%), UK (7.5%) and New Zealand (0.9%)	Beck Anxiety Inventory (BAI)	Eating Disorder Examination Questionnaire (EDE-Q)
Ranta et al. [64]	Finland	2 years	2070	56.4%	15.5 (0.4)	NR	Social Phobia Inventory (SPIN)	Questionnaires formulated according to the DSM-IV-TR Criteria
Robinson et al. [65]	8 European countries	5 years	1623	51.1%	14.5 (0.4)	NR	Development and Well-Being Assessment (DAWBA)	Development and Well-Being Assessment (DAWBA)
Schaumberg et al. [14]	UK	4 years and 6 years	7767	NR	10 (NR)	NR	Development and Wellbeing Assessment (DAWBA)	Youth Risk Behavior Surveillance System Questionnaire (YRBSSQ)
Schulte [66]	United Arab Emirates	5 months	236	NR	19.8 (1.45)	NR	Obsessive–Compulsive Inventory (OCI-R)	Binge Eating Scale (BES), & Emotional Eating Scale (EES)
Sherry et al. [67]	Canada	Daily EMA over 7 days	572	100%	19.5 (2.6)	90.8% were either Asian Canadian (N = 257) or European Canadian (N = 257)	POMS, cognitive worry subscale (CWS), autonomic emotional subscale (AES)	Bulimia Test‚ Revised
Sihvola et al. [68]	Finland	3 years	1852	49%	14.2 (NR)	NR	Semi-structured interview	Self-reported ED
Tanofsky-Kraff et al. [69]	US	4.7 years	195	NR	10.4 (1.5)	NR	State-Trait Anxiety Inventory for Children	Eating Disorder Examination & Standard Pediatric Eating Episode Interview
Trompeter et al. [70]	Australia	2 years, yearly assessments	2073	55%	13.8 (1.2)	89.9% born in Australia, 5.1% identified as Aboriginal and/or Torres Strait Islander	Brief Fear of Negative Evaluation (BFNE) scale	Eating Disorder Examination Questionnaire (EDE-Q)
van Eeden et al. [71]	Netherlands	Every 3 years	1881	50.8%	11.1 (0.6)	10.6% ethnic minority	Revised Child Anxiety and Depression Scale (RCADS)	Structured Clinical Interview for DSM Axis I disorders (SCID-I) & EDE at age 19; Eating Disorder Diagnostic Scale (EDDS) at ages 22 and 25
Verschueren et al. [72]	Belgium	2 years, annual assessments	2162	53.93%	14.6 (1.9)	NR	Cognitive Emotion Regulation Questionnaire (CERQ-short)	Eating Disorder Inventory-3 (EDI-3)
Webb et al. [73]	Australia	Every 6 months for 2 years	379	56%	12 (0.9)	White/Caucasian (80%), Asian (15%), Australian first peoples or Pacific Islander (1%), or range of other sociocultural backgrounds (4%)	Social Anxiety Scale for Children‚ Revised	Dutch Eating Behavior Questionnaire (DEBQ)
Zelkowitz et al. [74]	US	11 years	1420	51.1%	9–13 (range)	64.51% Caucasian, 5.56% African American, 0.21% Asian American, 0.42% Hispanic, and 22.75% American Indian	Child and Adolescent Psychiatric Assessment (CAPA)	Child and Adolescent Psychiatric Assessment (CAPA)
Zerwas et al. [75]	US	11 years	445	100%	NR	NR	Items from the Child Behavior Check List (CBCL)	Eating Attitudes Test-26 (EAT-26)

^1^For the daily diary studies, only data at Wave 1 (2019) was included due to acute COVID-19 pandemic at Wave 2. For the longitudinal comparison, both waves were included

**Table 2:** Quality assessment of included studies

First author, Years	Clear research question	Study population	Participation rate	Representative sample	Sample size justification	Exposure measured prior to outcome	Sufficient timeframe	Variability in exposure	Exposure clearly defined	Exposure assessed more than once	Outcome clearly defined	Outcome assessment blind to exposure	Loss to follow-up	Confounders	Rating
Abebe et al. [30]	Yes	Yes	Yes	Yes	No	Yes	Yes	Yes	Yes	Yes	Yes	NA	Yes	Yes	Good
Allen et al. [31]	Yes	Yes	Yes	Yes	Yes	Yes	Yes	Yes	Yes	Yes	Yes	NA	Yes	No	Good
Balantekin et al. [32]	Yes	No	NR	Yes	Yes	Yes	Yes	Yes	Yes	No	Yes	NA	Yes	Yes	Good
Bardone-Cone et al. [33]	Yes	No	NR	Yes	No	Yes	Yes	Yes	Yes	Yes	Yes	NA	No	No	Fair
Benjet [34]	Yes	Yes	Yes	Yes	Yes	Yes	Yes	NA	Yes	Yes	Yes	NR	No	Yes	Good
Bodell, et al. [35]	Yes	No	No	No	No	Yes	No	Yes	Yes	Yes	Yes	NA	Yes	No	Fair
Buckner et al. [18]	Yes	Yes	Yes	No	No	Yes	Yes	NA	Yes	Yes	Yes	NR	No	Yes	Good
Bufferd et al. [36]	Yes	No	No	Yes	No	Yes	Yes	Yes	Yes	No	Yes	NR	Yes	Yes	Fair
Cronce et al. [37]	Yes	No	No	Yes	No	Yes	Yes	Yes	Yes	No	No	NA	No	Yes	Fair
Dworschak, et al. [38]	Yes	Yes	No	Yes	No	Yes	Yes	Yes	Yes	Yes	No	NA	Yes	Yes	Good
Fairweather-Schmidt and Wade [39]	Yes	No	NR	Yes	No	Yes	Yes	Yes	Yes	No	Yes	NA	Yes	No	Fair
Fitzsimmons-Craft et al. [40]	Yes	No	No	Yes	Yes	Yes	Yes	Yes	Yes	Yes	Yes	NA	No	Yes	Fair
Fitzsimmons-Craft et al. [40]	Yes	No	No	Yes	No	Yes	Yes	Yes	Yes	Yes	Yes	NA	NR	No	Good
Gilbert and Meyer [41]	Yes	No	No	Yes	No	Yes	Yes	Yes	Yes	No	Yes	NA	No	Yes	Fair
Goodwin et al. [42]	Yes	No	NR	Yes	No	Yes	Yes	Yes	Yes	No	Yes	NA	NR	Yes	Fair
Hamann, et al. [43]	Yes	No	No	Yes	No	Yes	No	Yes	Yes	No	Yes	NA	NR	Yes	Fair
Hanback [44]	Yes	No	NR	Yes	No	Yes	Yes	Yes	Yes	Yes	Yes	NA	Yes	Yes	Good
Hautala et al. [45]	Yes	Yes	Yes	Yes	No	Yes	Yes	No	No	No	Yes	NA	NR	No	Fair
Herpertz-Dahlmann et al. [46]	Yes	Yes	Yes	No	No	Yes	Yes	Yes	Yes	No	Yes	NA	No	Yes	Good
Holm-Denoma and Hankin [47]	Yes	No	Yes	Yes	No	Yes	No	Yes	Yes	Yes	Yes	NA	Yes	Yes	Good
Hou et al. [48]	Yes	No	No	Yes	No	Yes	Yes	Yes	Yes	Yes	Yes	NA	No	Yes	Good
Isaksson [49]	No	No	NR	Yes	Yes	Yes	Yes	Yes	Yes	Yes	No	NA	No	Yes	Good
Johnson et al. [50]	Yes	No	NR	Yes	No	Yes	Yes	Yes	Yes	Yes	Yes	NR	NR	Yes	Fair
Kidwell et al. [51]	Yes	No	NR	Yes	Yes	Yes	Yes	Yes	Yes	No	Yes	NA	Yes	Yes	Good
Lacroix et al. [52]	Yes	No	Yes	Yes	No	Yes	Yes	Yes	Yes	No	Yes	No	Yes	Yes	Good
Le Grange et al. [23]	Yes	Yes	NR	Yes	No	Yes	Yes	No	No	No	Yes	NA	Yes	Yes	Fair
Lee and Vaillancourt [53]	Yes	Yes	No	Yes	No	Yes	Yes	Yes	Yes	Yes	Yes	NA	Yes	Yes	Good
Levinson and Sala (2016) [29]	Yes	No	No	Yes	No	Yes	Yes	Yes	Yes	Yes	Yes	NA	No	Yes	Fair
Lieb et al. [54]	Yes	Yes	Yes	Yes	No	Yes	Yes	NA	Yes	No	Yes	NR	Yes	Yes	Good
Lim et al. [55]	Yes	No	No	Yes	Yes	Yes	Yes	Yes	Yes	Yes	Yes	NA	Yes	Yes	Good
Linardon et al. [24]	Yes	No	Yes	Yes	No	Yes	Yes	Yes	Yes	Yes	Yes	NA	No	Yes	Good
Lloyd et al. [9]	Yes	Yes	Yes	Yes	Yes	Yes	Yes	Yes	Yes	Yes	Yes	NA	Yes	Yes	Good
Loose et al. [56]	Yes	Yes	Yes	Yes	Yes	Yes	Yes	No	Yes	Yes	Yes	NA	NR	Yes	Good
Magson et al. [57]	Yes	No	No	Yes	Yes	Yes	Yes	Yes	Yes	Yes	Yes	NA	Yes	No	Good
McLaughlin et al. [58]	Yes	No	No	Yes	No	Yes	Yes	Yes	Yes	No	Yes	NA	Yes	Yes	Good
Micali et al. [17]	Yes	Yes	Yes	Yes	Yes	Yes	Yes	NA	Yes	Yes	Yes	NA	Yes	Yes	Good
Minnich et al. [59]	Yes	No	No	No	No	Yes	No	Yes	Yes	Yes	No	NA	Yes	No	Fair
Nolen-Hoeksema et al. [60]	Yes	No	Yes	NR	No	Yes	Yes	Yes	Yes	Yes	Yes	NR	Yes	Yes	Fair
Parker et al. [61]	Yes	Yes	No	NR	Yes	Yes	Yes	Yes	Yes	Yes	No	NA	No	Yes	Good
Patton et al. [62]	Yes	Yes	No	Yes	No	Yes	Yes	No	Yes	Yes	Yes	NA	Yes	No	Good
Puccio et al. [63]	Yes	No	No	Yes	Yes	Yes	Yes	Yes	Yes	Yes	Yes	NA	NR	Yes	Good
Ranta et al. [64]	Yes	Yes	Yes	Yes	No	Yes	Yes	No	Yes	Yes	No	NA	Yes	Yes	Good
Robinson et al. [65]	Yes	Yes	NR	Yes	No	Yes	Yes	No	Yes	Yes	Yes	NA	No	Yes	Good
Schaumberg et al. [14]	Yes	Yes	Yes	Yes	No	Yes	Yes	Yes	Yes	No	Yes	NA	No	Yes	Good
Schulte [66]	Yes	No	No	Yes	No	Yes	No	Yes	Yes	No	Yes	NA	No	Yes	Fair
Sherry et al. [67]	Yes	No	No	Yes	No	Yes	Yes	Yes	Yes	Yes	Yes	NA	Yes	Yes	Good
Sihvola et al. [68]	Yes	Yes	Yes	No	No	Yes	Yes	NA	Yes	No	No	NA	Yes	Yes	Good
Tanofsky-Kraffat al. [69]	Yes	No	No	Yes	No	Yes	Yes	Yes	Yes	No	Yes	NR	NR	Yes	Fair
Trompeter et al. [70]	Yes	Yes	No	Yes	Yes	Yes	Yes	Yes	Yes	Yes	Yes	NA	No	Yes	Good
van Eeden et al. [71]	Yes	Yes	Yes	Yes	Yes	Yes	Yes	Yes	Yes	No	Yes	NA	Yes	Yes	Good
Verschueren et al. [72]	Yes	Yes	No	Yes	No	Yes	Yes	Yes	Yes	Yes	Yes	NA	Yes	Yes	Good
Webb et al. [73]	Yes	No	No	No	No	Yes	Yes	Yes	Yes	No	Yes	NA	NR	Yes	Fair
Zelkowitz et al. [74]	Yes	Yes	NR	No	Yes	Yes	Yes	No	No	Yes	Yes	NA	No	Yes	Fair
Zerwas, (2014) [75]	Yes	No	No	NR	No	Yes	Yes	Yes	Yes	Yes	Yes	NA	No	Yes	Good

*Note*: *Yes* criteria met, *No* criteria not met, *CD* can’t be determined, *NA* not applicable, *NR* not reported

## Findings: anxiety symptoms predicting eating disorder symptoms

In total, 47 of the 54 studies examined the prospective association between anxiety symptoms and eating disorder symptoms. A majority of studies (24 of 46, 51.1%) found evidence for a link between anxiety symptoms and subsequent eating disorder symptoms, with some studies (16 of 47, 34.0%) reporting mixed findings, and few studies (7 of 47, 14.9%) reporting no significant association. Studies varied greatly in terms of age ranges covered (baseline age range: 3–20.9 years), and the domains of anxiety or eating disorder measured. In terms of gender, there were 26 mixed gender studies, 15 female-only studies, 1 male-only study, and 5 not reported.

### *Meta*-analysis 1: Anxiety symptoms predicting eating disorder symptoms (dichotomous)

**Fig. 2:**
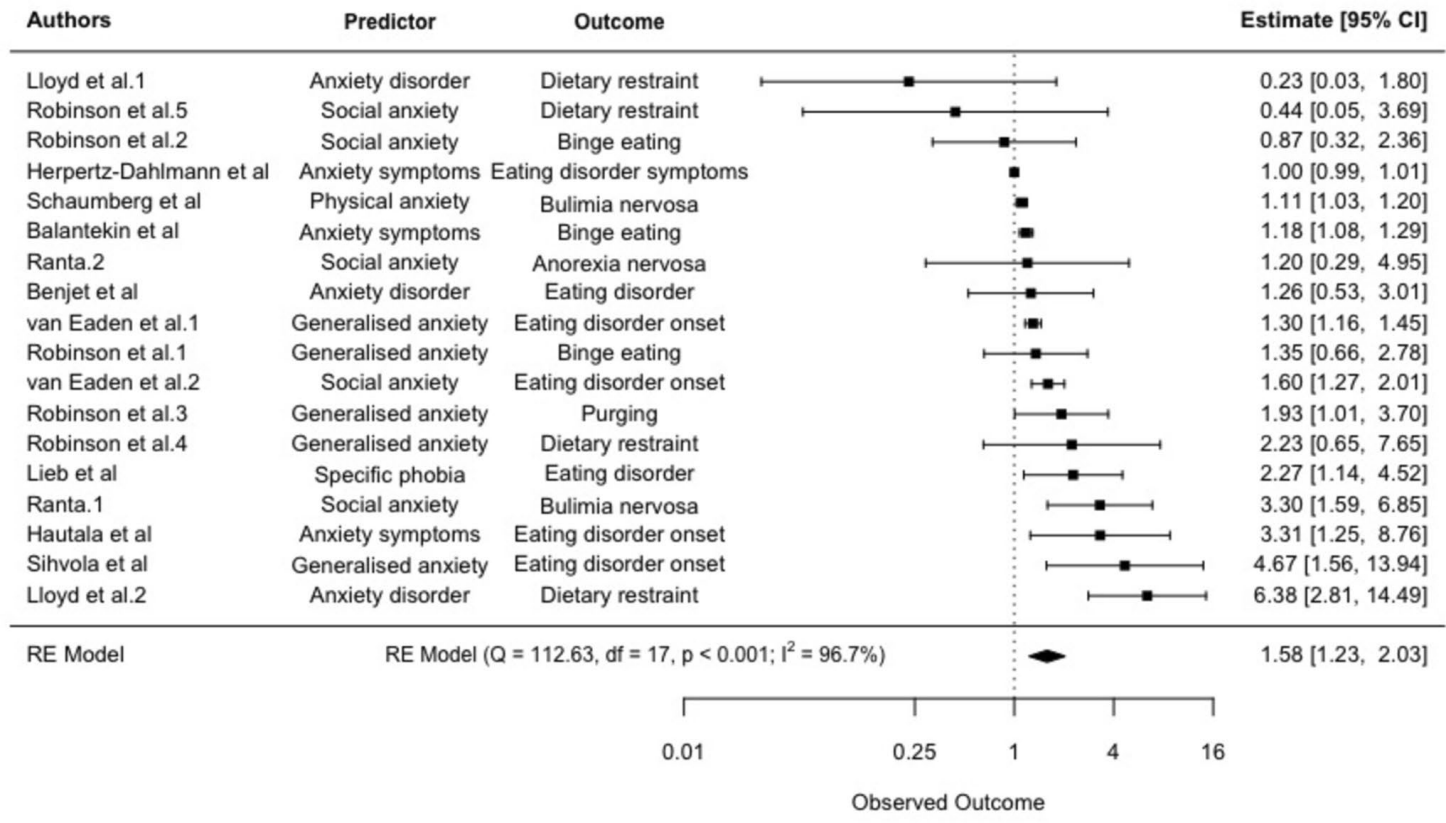
Random effects meta-analysis: Forest plots comparing odds ratios for anxiety symptoms and subsequent eating disorder symptoms (95% CI). Observed outcome = log odds. Estimate = Odds ratio

Eleven studies reported on 21 separate effect sizes investigating the relationship between anxiety symptoms and subsequent eating disorder symptoms using dichotomous measures. Having anxiety symptoms was associated with significantly higher odds of subsequent eating disorder symptoms, with a pooled effect size of OR = 1.58 (95% CI: 1.23–2.03, *p* < 0.001), see Fig. 2. Heterogeneity was significant and high (Q = 112.63, *p* < 0.001; *I*^2^ = 96.70%). Egger's regression test showed significant funnel plot asymmetry (*z* = 2.76, *p* = 0.006). Age of participants at baseline was a significant moderator accounting for 28.23% of heterogeneity (tau^2^ = 0.13, *SE* = 0.08; test for residual heterogeneity: QE(*df* = 15) = 81.76, *p* < 0.001; test of moderators: QM(*df* = 1) = 4.01, *p* = 0.045), whereby the relationship between anxiety symptoms and subsequent eating disorder symptoms was stronger for studies with older participants at baseline. Length of follow-up was not a significant moderator (tau^2^ = 0.15, *SE* = 0.09; test for residual heterogeneity: QE(*df* = 16) = 91.86, *p* < 0.001; test of moderators: QM(*df* = 1) = 1.92, *p* = 0.166).

Sensitivity analysis using a multi-level meta-analysis accounting for study dependence found a similar relationship, with a pooled effect size of OR = 1.58 (95% CI: 1.21–2.07, *p* = 0.002).

### *Meta*-analysis 2: Anxiety symptoms predicting eating disorder symptoms (continuous)

**Fig. 3:**
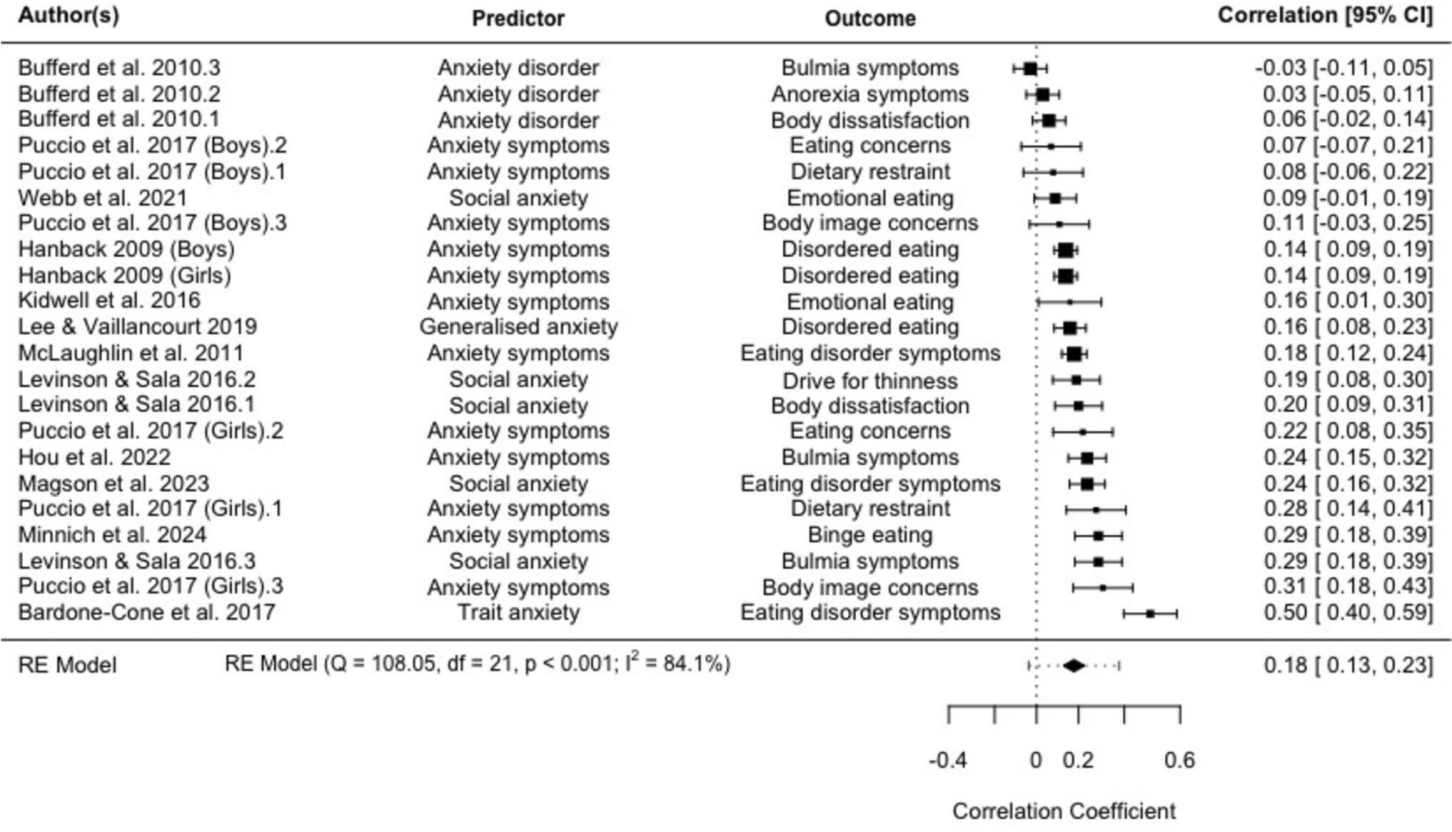
Random effects meta-analysis: Forest plots comparing correlation coefficients for anxiety symptoms and subsequent eating disorder symptoms (95% CI)

Twelve studies reported on 22 separate effect sizes investigating the relationship between anxiety symptoms and subsequent eating disorder symptoms using continuous measures. Anxiety symptoms were associated with significantly higher subsequent eating disorder symptoms, with a pooled effect size of *r* = 0.18 (95% CI: 0.13–0.23, *p* < 0.001), see Fig. 3. Heterogeneity was significant and high (Q = 108.05, *p* < 0.001; *I*^2^ = 84.09%). Egger's regression test showed no significant funnel plot asymmetry (*z* = 1.52, *p* = 0.130). Age of participants at baseline was a significant moderator accounting for 58.54% of heterogeneity (tau^2^ = 0.01, *SE* = 0.00; test for residual heterogeneity: QE(*df* = 18) = 55.07, *p* < 0.001; test of moderators: QM(*df* = 1) = 17.05, *p* < 0.001), whereby the relationship between anxiety symptoms and subsequent eating disorder symptoms was stronger for studies with older participants at baseline. Length of follow-up was a significant moderator accounting for 43.52% of heterogeneity (tau^2^ = 0.01, *SE* = 0.00; test for residual heterogeneity: QE(*df* = 20) = 66.15, *p* < 0.001; test of moderators: QM(*df* = 1) = 10.98 *p* < 0.001), whereby the relationship between anxiety symptoms and subsequent eating disorder symptoms was stronger for studies with shorter delays.

Sensitivity analysis using a multi-level meta-analysis accounting for study dependence found a similar relationship, with a pooled effect size of *r* = 0.20 (95% CI: 0.13–0.27, *p* < 0.001).

### Narrative synthesis: Childhood anxiety to adolescent eating disorder symptoms

Eight studies reported on the association between childhood anxiety symptoms and eating disorder symptoms in adolescence. Of these, five found significant associations, two mixed findings, and one did not find a significant association between childhood anxiety symptoms and adolescent eating disorder symptoms.

Most studies focused on general anxiety symptoms. These studies found that childhood anxiety symptoms predicted subsequent binge eating [14], fasting [14], emotional eating [14, 51], body image concerns [14, 36], and overall disordered eating [75] in adolescence. However, there were mixed findings around eating disorder diagnosis. Buffered et al. [36] found that meeting criteria for an anxiety disorder in childhood was not associated with meeting criteria for either anorexia nervosa or bulimia nervosa. Similarly, Tanofsky-Kraff et al. [69] found that anxiety at age 10 did not predict onset of binge eating disorder. Mixed findings were reported by Schaumberg et al. [14], who found that physical symptoms of anxiety at age 10 predicted higher odds of meeting criteria for bulimia nervosa at age 16, but not for anorexia nervosa. In contrast, van Eeden et al. [71] found that anxiety disorders in childhood, especially generalised anxiety disorder or social anxiety disorder, predicted having an eating disorder in late adolescence. Regarding specific symptoms, Dworschak et al. [38] reported that rumination in a mixed child/adolescent sample (ages 8–15) was related to more frequent weight concerns and more frequent restrictive eating at the person-level (i.e., more frequent rumination than normal was associated with more frequent weight concerns and restrictive eating than normal) over a 21-day period. Additionally, more frequent rumination predicted higher frequency of restrictive behaviours, but not weight concerns one year later.

Taken together, the reviewed evidence suggests that childhood anxiety is linked with broad eating disorder pathology in adolescence. More research on whether this extends to an increased risk for meeting criteria for an eating disorder is required.

### Narrative synthesis: Adolescent anxiety to adolescent eating disorder symptoms

Most studies (33 of 46, 69.6%) reported on the association between anxiety symptoms and subsequent eating disorder symptoms in adolescence. Of these, 17 (51.5%) found significant associations, 11 (33.3%) mixed findings, and 5 (15.2%) did not find a significant association between anxiety symptoms and subsequent eating disorder symptoms in adolescence.

Overall, anxiety symptoms were found to predict both subsequent eating disorder symptoms, including general eating pathology [33, 45, 58], bulimic symptoms [48], binge eating [59], and fasting [25], and increases in eating disorder symptoms, such as bulimic symptoms [48], general eating pathology [53] and weight control behaviours in girls only [44]. In particular, Lacroix et al. [52] showed that trait anxiety at age 11 predicted membership of a high-risk eating disorder trajectory throughout adolescence and year adulthood compared to a low-risk membership. However, findings did not seem to hold in studies controlling for depression [23, 35, 41, 63]. Further, no significant relationship was found when looking at daily anxious affect predicting binge eating [67]. Mixed findings were also evident when distinguishing between-person from within-person changes. Fitzsimmons-Craft et al. [40] found that while anxiety predicted both between-person differences in dietary restraint and binge eating, anxiety only predicted within-person binge eating and not dietary restraint in a university sample (i.e., emerging adults). However, among early adolescents Parker et al. [61] found anxiety to only predict between-person differences in loss of control eating and not within-person changes. Focusing on within-person changes, Lim et al. [55] reported no significant findings for binge eating, uncontrolled eating, or emotional eating, but did find a significant stress and anxiety interaction that predicted within-person changes in dietary restraint. That is, both lower than usual stress and anxiety predicted greater than usual increases in dietary restraint.

Symptoms of specific anxiety disorders showed mixed findings for social anxiety symptoms. Social anxiety symptoms were positively related to subsequent eating disorder symptoms, such as bulimic symptoms [64], eating disorder pathology [57], binge eating [65], emotional eating [73], and dietary restraint [65], but not subsequent symptoms of anorexia nervosa [64], or purging [65]. Further, there was no evidence that social anxiety predicted increases in eating disorder symptoms (Levinson & Sala, et al., 2016; 64]. A similar pattern was evident for symptoms of generalised anxiety. Findings showed that generalised anxiety predicted subsequent eating disorder diagnosis [68], dietary restraint [65], purging [65], and binge eating [65]. However, there were mixed findings when predicting increases in eating disorder symptoms. Levinson and Sala, et al. (2016) found that generalised anxiety predicted increases in drive for thinness, but not bulimic symptoms or body image concerns. Limited studies examined other anxiety disorders. Of these, OCD symptoms were found to predict increases in driven exercise among boys, but not girls [42], and predicted subsequent binge eating, but not increases in binge eating [66]. Further, PTSD symptoms predicted increases in binge eating [37], eating disorder thoughts [49] and compensatory behaviours [49].

Turning to more mechanistic research, a limited number of studies investigated specific anxiety symptoms. Among these, two key symptoms were examined in the literature: fear of negative evaluation, a core feature of social anxiety, and rumination, a core feature of generalised anxiety. There were mixed findings regarding fear of negative evaluation. Two studies found that fear of negative evaluation predicted increases in bulimic symptoms [41, 43], whereas one did not (Levinson & Sala, et al., 2016). All three were conducted among undergraduate women (i.e., emerging adults). Further, two studies found that among undergraduate women fear of negative evaluation did not predict increases in body image concerns [41, 70]. No gender differences were found in the associations. Regarding rumination, there appears to be evidence implicating rumination in bulimic symptoms. Findings show that rumination predicted both subsequent bulimic symptoms [47], and increases in bulimic symptoms [60, 72]. More specifically, rumination was shown to predict the onset of binge eating, but not compensatory behaviours [60]. Further, rumination was shown not to be related to subsequent overall eating symptoms [58].

Taken together, the reviewed evidence suggests that anxiety is linked with subsequent eating disorder pathology in adolescence, however, this association is not independent of depression. Further research is also required to distinguish between-person changes from within-person changes in the prospective relationship between anxiety and eating disorder symptoms. Of note, few studies investigated specific anxiety symptoms that may be implicated in the relationship between anxiety and eating disorder symptoms. Within these limited studies, there is converging evidence that rumination is linked to bulimic symptoms, but more work is needed regarding fear of negative evaluation.

### Narrative synthesis: Adolescent anxiety to adult eating disorder symptoms

Six studies reported on the association between adolescent anxiety symptoms and eating disorder symptoms in adulthood. Of these, two found significant associations, three mixed findings, and one did not find a significant association between anxiety symptoms and eating disorder symptoms in adulthood.

Most studies focused on associations at a disorder-level and found that associations may be disorder-dependent. When looking at disorders overall, no association was found between meeting criteria for an anxiety disorder in adolescence and meeting criteria for an eating disorder in adulthood [34]. More nuanced findings emerged when looking by criteria. Meeting criteria for OCD in adolescence predicted the onset of anorexia nervosa in adulthood [18], and PTSD symptoms in adolescence predicted the onset of bulimia nervosa in adulthood [74]. Meeting criteria for a specific phobia predicted increased odds of meeting criteria for an eating disorder [54], but not onset of either anorexia nervosa or bulimia nervosa [18]. No significant findings were noted for social anxiety or generalised anxiety [18]. At a symptom level, overall anxiety predicted subsequent eating pathology [46], and increases in bulimic symptoms [30], but not overall eating pathology [46].

Taken together, the reviewed evidence suggests that there is a tentative link between adolescent anxiety and eating disorder symptoms in adulthood that requires further research. Of note, most studies focused on diagnostic groups rather than overall anxiety which may hinder detection of significant associations.

### Conclusion: Anxiety symptoms predicting eating disorder symptoms

In conclusion, the reviewed evidence suggests that there is a link between anxiety and eating disorder symptoms that requires further research. Findings from the meta-analysis show that symptoms of anxiety are associated with higher odds of experiencing eating disorder symptoms and with high levels of eating disorder symptoms in the future. However, effect sizes were small, and studies were highly heterogenous. The narrative synthesis further showed that anxiety symptoms may predict the onset of eating disorder symptoms, as well as the increase in symptoms. However, more research is needed to further elucidate this relationship. For example, few studies examined gender differences or differentiated within-person from between-person associations.

## Findings: Eating disorder symptoms predicting anxiety symptoms

In total, 25 of the 54 studies examined the prospective association between eating disorder symptoms and subsequent anxiety symptoms. Most studies (15 of 25, 60.0%) found evidence for a link, with some studies (7 of 25, 28.0%) reporting mixed findings, and few studies (3 of 25, 12%) reporting no significant association. Studies varied greatly in terms of age ranges covered (baseline age range: 10.4–19.8 years), and the domains of anxiety or eating disorder measured. In terms of gender, there were 15 mixed gender studies, 5 female-only studies, 1 male-only study, and 4 not reported.

### *Meta*-analysis 3: Eating disorder symptoms predicting anxiety symptoms (dichotomous)

**Fig. 4:**
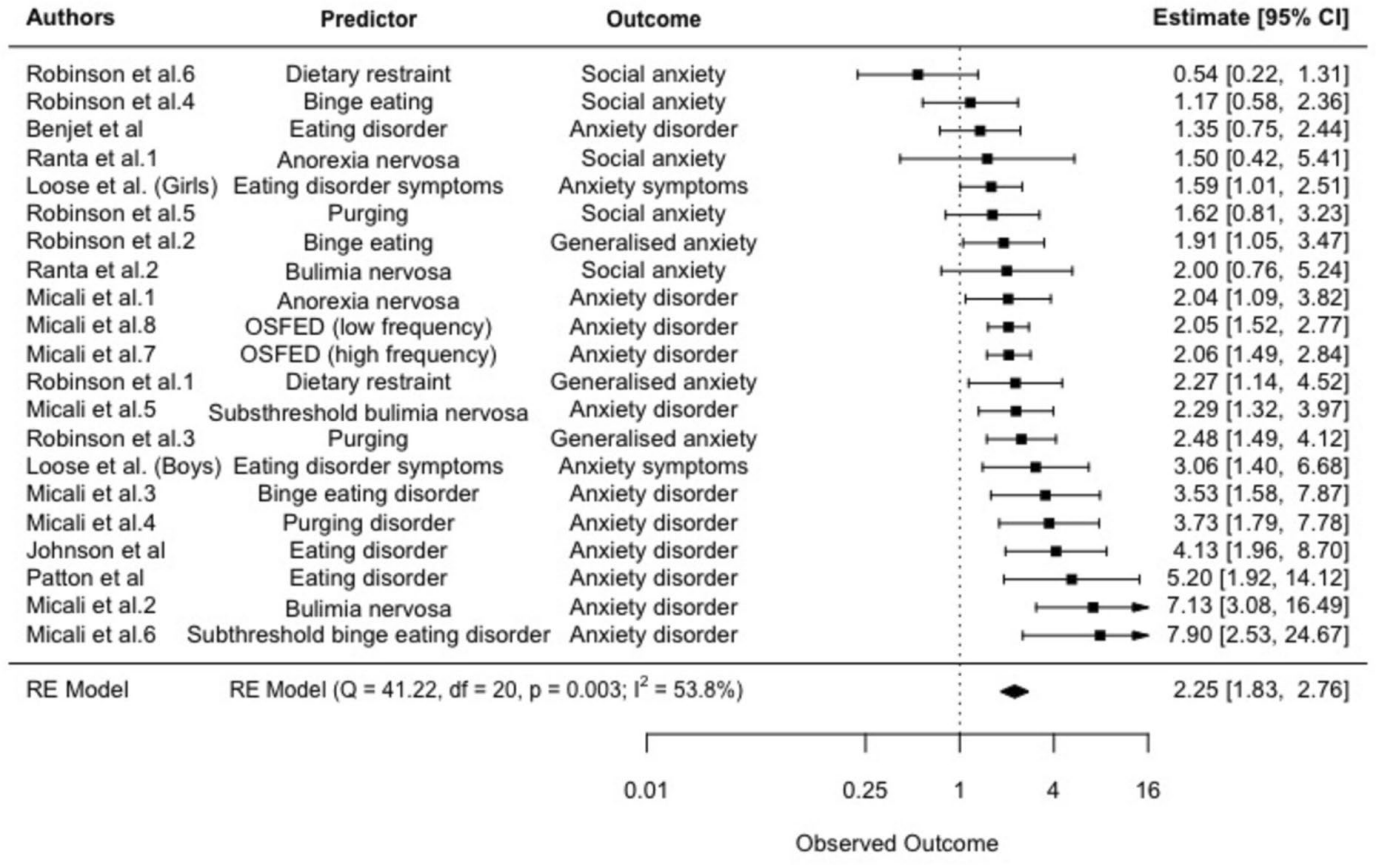
Random effects meta-analysis: Forest plots comparing odds ratios for eating disorder symptoms and subsequent anxiety symptoms (95% CI). Observed outcome = log odds. Estimate = Odds ratio

Seven studies reported on 21 separate effect sizes investigating the relationship between eating disorder symptoms and subsequent anxiety symptoms using dichotomous measures. Having eating disorder symptoms was associated with significantly higher odds of subsequent anxiety symptoms, with a pooled effect size of OR = 2.25 (95% CI: 1.83–2.76, *p* < 0.001), see Fig. 4. Heterogeneity was significant and moderate (Q = 41.22, *p* = 0.004; *I*^2^ = 53.80%). Egger's regression test showed no significant funnel plot asymmetry (*z* = 1.34, *p* = 0.179). Neither age of participants at baseline (tau^2^ = 0.13, *SE* = 0.08; test for residual heterogeneity: QE(*df* = 18) = 38.76, *p* = 0.003; test of moderators: QM(*df* = 1) = 0.23, *p* = 0.628) or length of follow-up were significant moderators (tau^2^ = 0.15, *SE* = 0.09; test for residual heterogeneity: QE(*df* = 18) = 20.27, *p* = 0.002; test of moderators: QM(*df* = 1) = 0.36, *p* = 0.551).

Sensitivity analysis using a multi-level meta-analysis accounting for study dependence found a similar relationship, with a pooled effect size of OR = 2.21 (95% CI: 1.64–2.99, *p* < 0.001).

### *Meta*-analysis 4: Eating disorder symptoms predicting anxiety symptoms (continuous)

**Fig. 5:**
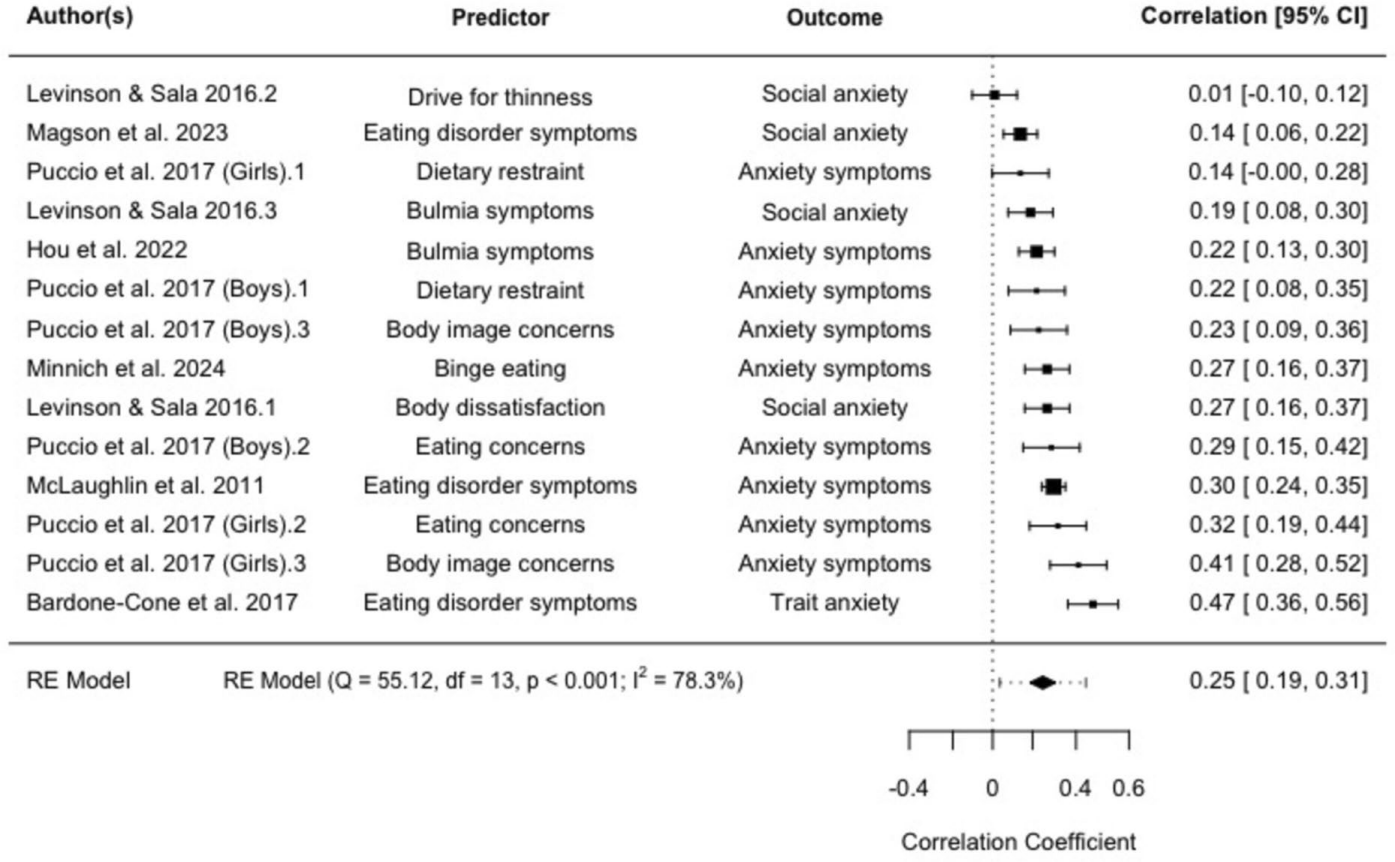
Random effects meta-analysis: Forest plots comparing correlations for eating disorder symptoms and subsequent anxiety symptoms (95% CI)

Seven studies reported on 14 separate effect sizes investigating the relationship between eating disorder symptoms and subsequent anxiety symptoms using continuous measures. Eating disorder symptoms were associated with significantly higher subsequent anxiety symptoms, with a pooled effect size of *r* = 0.25 (95% CI: 0.19–0.32, *p* < 0.001), see Fig. 5. Heterogeneity was significant and high (Q = 55.12, *p* < 0.001; *I*^2^ = 78.26%). Egger's regression test showed no significant funnel plot asymmetry (*z* = 0.64, *p* = 0.520). Neither age of participants at baseline (tau^2^ = 0.01, *SE* = 0.01; test for residual heterogeneity: QE(*df* = 11) = 47.97, *p* < 0.001; test of moderators: QM(*df* = 1) = 0.29, *p* = 0.590) or length of follow-up were significant moderators (tau^2^ = 0.01, *SE* = 0.01; test for residual heterogeneity: QE(*df* = 12) = 53.46, *p* < 0.001; test of moderators: QM(*df* = 1) = 0.01, *p* = 0.916).

Sensitivity analysis using a multi-level meta-analysis accounting for study dependence found a similar relationship, with a pooled effect size of *r* = 0.26 (95% CI: 0.18–0.33, *p* < 0.001).

### Narrative synthesis: Adolescent eating disorder symptoms to adolescent anxiety symptoms

Most studies (19 of 25) reported on the association between eating disorder symptoms and subsequent anxiety symptoms in adolescence. Of these, 12 found significant associations, 5 mixed findings, and 2 did not find a significant association between eating disorder symptoms and subsequent anxiety symptoms in adolescence.

Overall eating pathology in adolescence predicted subsequent overall anxiety symptoms [31, 33, 58], social anxiety [57], and rumination [58]. Meeting criteria for any eating disorder in adolescence predicted higher odds of meeting criteria for an anxiety disorder [17], but not subsequent social anxiety [64].

Turning to specific eating disorder symptoms, body image concerns predicted subsequent anxiety symptoms [39] as well as increases in anxiety symptoms [63] and fear of negative evaluation [70]. Body image concerns did not predict subsequent anxiety in late adolescent boys [59], or increases in worry in late adolescent girls (Levinson & Sala, et al., 2016). Bulimic symptoms predicted subsequent rumination [47], as well as increased overall anxiety [48] and increased rumination [60, 72]. However, bulimic symptoms did not predict increases in worry (Levinson & Sala, et al., 2016). Binge eating predicted subsequent overall anxiety [59, 69], OCD symptoms [66], and increased anxiety [69], but not onset of anxiety [65]. When distinguishing between and within-person associations, there was a significant between-person associations between loss of control eating and subsequent anxiety, but not within-person association [61]. Mixed findings emerged for dietary restraint. Dietary restraint predicted the onset of generalised anxiety but not social anxiety [65], nor did it predict subsequent overall anxiety or increases in overall anxiety [63]. Only one study examined purging, whereby purging predicted the onset of generalised anxiety but not social anxiety [65]. No study examined driven exercise specifically.

Taken together, the reviewed evidence supports a link between eating disorder symptoms and subsequent anxiety symptoms in adolescence. Further, evidence suggests that eating disorder symptoms predict increases in anxiety symptoms. In particular, overall eating pathology appears to be related to subsequent anxiety symptoms. More research is needed to understand within-person and between-person association, as well as specific symptom associations.

### Narrative synthesis: Adolescent eating disorder symptoms to adult anxiety symptoms

Six studies reported on the association between adolescent eating disorder symptoms and anxiety symptoms in adulthood. Of these, three found significant associations, two mixed findings, and one did not find a significant association between adolescent eating disorder symptoms and anxiety symptoms in adulthood.

Most studies examined the association at a diagnostic-level and found mixed results. Two studies found a significant association between meeting criteria for an eating disorder in adolescence and meeting criteria for an anxiety disorder in adulthood [50, 62], whereas another did not [34]. When looking at specific diagnoses, meeting criteria for anorexia nervosa in adolescence did not predict meeting criteria for any anxiety disorder in adulthood, but meeting criteria for bulimia nervosa in adolescence precited the onset of panic disorder and social anxiety disorder [18]. At a more symptomatic level, overall eating disorder symptoms predicted subsequent generalised anxiety in adulthood, and increased generalised anxiety in men but not women [56]. Similarly, both bulimic symptoms and body image concerns in adolescence predicted subsequent overall anxiety in both men and women, but the relationship between bulimic symptoms and subsequent anxiety was stronger in men compared to women.

Taken together, the reviewed evidence supports a link between eating disorder symptoms in adolescence and anxiety symptoms in adulthood. More research is needed to examine potential gender differences, and differences by diagnoses.

### Conclusion: Eating symptoms predicting anxiety symptoms

In conclusion, the reviewed evidence suggests that there is a link between anxiety and eating disorder symptoms. Findings from the meta-analysis show that symptoms of eating disorder are associated with higher odds of experiencing anxiety symptoms in the future and higher levels of anxiety symptoms. However, effect sizes were small, and studies were highly heterogenous. The narrative synthesis further showed that anxiety symptoms may predict an increase in symptoms. However, more research is needed to further elucidate this relationship. For example, few studies examined gender differences or differentiated within-person from between-person associations.

## Findings: Bi-directional relationship between anxiety and eating disorder symptoms

In total, 6 of the 54 studies specifically examined the prospective bi-directional association between anxiety and eating disorder symptoms. Of these, four found evidence for a bi-directional relationship whereas two did not.

Specifically, Holm found a bi-directional relationship between rumination and bulimic symptoms, whereby rumination predicted increased bulimic symptoms and bulimic symptoms predicted increased rumination. The same pattern was observed by Nolen et al. in adolescent girls. Similarly, Hou found a bi-directional relationship between overall anxiety and bulimic symptoms, whereby anxiety symptoms predicted increased bulimic symptoms and bulimic symptoms predicted increased anxiety symptoms. However, in their cross-lag panel model the first path of bulimic symptoms predicting subsequent anxiety symptoms was not significant, suggesting that the bi-directional nature of the association is established only after increases in anxiety symptoms are experienced. In contrast, Levinson & Sala, et al. (2016) found no evidence of a bi-directional relationship when examining bulimic symptoms among other eating disorder symptoms in young women. In their study, worry predicted increases in drive for thinness, but not body dissatisfaction or bulimic symptoms. No links between eating disorder symptoms and subsequent worry were found.

Regarding other eating disorder symptoms, Trompeter found a bi-directional relationship between fear of negative evaluation and body image concerns [41], whereby fear of negative evaluation predicted increased body image concerns and body image concerns predicted increased fear of negative evaluation. However, Puccio found no evidence of a bi-directional relationship when examining body image concerns among other symptoms. Instead, findings showed that both body image and eating concerns predicted increased anxiety symptoms during late adolescence, but not vice versa. Interestingly, no significant associations were found earlier in adolescence or for dietary restraint, a behavioural eating disorder symptom. Of note, unlike other studies, this study controlled for depression which may account for some discrepancies between results.

In conclusion, there is some evidence that anxiety and eating disorder symptoms exhibit a bi-directional relationship, especially concerning rumination and bulimic symptoms. However, more research needs to test this relationship explicitly.

## Discussion

The current review synthesised evidence from 54 studies to examine the prospective relationship between anxiety symptoms and eating disorder symptoms in adolescence, including four meta-analyses. Overall, evidence suggests that there is a prospective and bi-directional relationship between anxiety symptoms and eating disorder symptoms. Anxiety symptoms were shown to be associated with subsequent eating disorder symptoms, increases in eating disorder symptoms, and higher odds of eating disorders, including the onset of such disorders. Conversely, eating disorder symptoms were also associated with subsequent anxiety symptoms, increases in anxiety symptoms, and higher odds of anxiety disorders.

Findings are in line with previous reviews highlighting a high co-morbidity between anxiety disorders and eating disorders [4, 76], showing that this extends beyond clinical samples and is prospective in nature. Extending findings to both community samples and prospective designs is critical in further developing our understanding of the co-morbidity between anxiety disorders and eating disorders. However, whilst findings from the current review show a clear bi-directional relationship between anxiety symptoms and eating disorder symptoms, the longitudinal relationship between these is less clear. For example, there is some evidence to suggest that anxiety symptoms might first be associated with eating disorder symptoms, after which time, a bi-directional relationship becomes evident. That is, in the absence of eating disorder symptoms, anxiety symptoms are linked to later onset of eating disorder symptoms [54, 71]. Further, Hou et al. [48] found that in a cross-lag panel model, anxiety symptoms were associated with subsequent bulimic symptoms across timepoints (i.e., from T1 to T2, and T2 to T3), whereas bulimic symptoms were only associated with anxiety symptoms at later timepoints (i.e., not from T1 to T2, but from T2 to T3). As such, anxiety symptoms may trigger eating disorder symptoms, which then maintain and potentially exacerbate existing anxiety symptoms. However, this could in part be due to the respective age of onset of anxiety disorders and eating disorders. While anxiety disorders typically have an onset in childhood, eating disorder frequently develop in adolescence or early adulthood [19]. Thus, anxiety symptoms may be early signs of later eating disorders rather than being causally related to the onset of symptoms. There is a genetic correlation between anxiety and anorexia nervosa [77], therefore the bi-directional relationship between anxiety symptoms and eating disorder symptoms could point towards a shared vulnerability factor, such as genetic predisposition [7, 78]. However, this research should be extended to clarify shared and specific genetic vulnerabilities. Further research should examine in detail the temporal relationship between anxiety symptoms and eating disorder symptoms to understand the mechanisms implicated in their relationship. Additionally, limited research has examined individual differences in symptom trajectories or identified different pathways through which symptoms are related to one another. Thus, further exploration of how anxiety and eating disorder symptoms relate to one another and how this varies between individuals is required.

## Research implications

Our review suggests several avenues for future research. Firstly, few studies differentiated between-person from within-person changes. Of the studies that did differentiate, most found significant between-person changes but only limited within-person changes. However, studies were limited with small sample sizes. Thus, more research is needed to distinguish these differences and determine whether associations exist at both a between-person and within-person level, or purely on a between-person level. This distinction is important to advance our understanding of the underlying mechanisms linking anxiety and eating disorder symptoms and improve early intervention and prevention methods. The current understanding from the reviewed evidence shows that adolescents with high anxiety symptoms (compared to others) are at risk for increased eating disorder symptoms (and vice versa; a between-person relationship), however it is still unclear whether adolescents who experience higher levels of anxiety than they would usually experience are also at risk of increased eating disorder symptoms and vice-versa (i.e., a within-person relationship). If only a between-person relationship existed, this would imply that adolescents with heightened anxiety symptoms can effectively be identified as being “at risk” for future eating disorder symptoms, and vice versa, but targeting these symptoms may not decrease this risk. However, if a within-person relationship existed, targeting existing levels of anxiety and/or eating disorder symptoms would likely reduce the risk of future anxiety and/or eating disorder symptoms.

Secondly, more research on mechanisms underlying the relationship between anxiety symptoms and eating disorder symptoms, and vice versa is needed. Evidence from this review suggests, that rumination may be particularly implicated in the development and maintenance of bulimic symptoms [47, 60, 72], however, more research is needed to support this link. Further, mixed evidence was evident for fear of negative evaluation as a specific mechanism. Studies with late adolescent girls found no significant relationship between fear of negative evaluation and body image concerns (Gilbert and Meyer [41]; Levinson & Sala, et al., 2016), whereas research among mixed-gender mid-adolescents found a significant bi-directional relationship [70]. Relatedly, few studies investigated gender differences and many studies were conducted in female-only samples. Of the studies that did investigate gender differences, there were mixed findings with evidence pointing towards a stronger relationship amongst boys. For example, Linardon et al. [24] found that the relationship between bulimic symptoms and subsequent anxiety was stronger for boys compared to girls. Loose et al. [56] found that while eating disorder symptoms predicted subsequent generalised anxiety symptoms in both boys and girls, increases in generalised anxiety symptoms (i.e., controlling for baseline levels) were only significant for boys. Lastly, Goodwin et al. [42] found that OCD symptoms predicted increases in excessive exercise among boys, but not girls. However, some studies found no gender differences in associations between anxiety symptoms and eating disorder symptoms, and vice versa [70, 72]. Thus, more research is needed to understand potential gender differences.

## Clinical implications

Findings from this review provide important clinical implications, particularly concerning prevention and early intervention for both anxiety and eating disorder symptoms. Understanding the prospective relationship between anxiety and eating disorder symptoms may assist the early detection of either anxiety symptoms in those with eating disorder symptoms or eating disorder symptoms in those with anxiety symptoms. Further, the bi-directional nature between anxiety and eating disorder symptoms may point towards a vicious cycle, in which symptoms may maintain one another. As such, effective treatments for adolescents with co-occurring anxiety symptoms and eating disorder symptoms should target both groups of symptoms, whether this is within the context of anxiety disorder or eating disorder treatment. While current treatments are likely to target some overlapping features (e.g., fear of weight gain in eating disorders, or appearance anxiety in anxiety disorders), to our knowledge there are not many specific treatments that target both anxiety and eating disorders, nor specific prevention programs. Greater focus on overlapping mechanisms (e.g., avoidance) may be beneficial both for prevention and treatment (see [8] for review).

## Limitations

This study has some limitations which should be considered when interpreting our results. Firstly, there were a limited number of studies in the meta-analyses, with high levels of heterogeneity. Secondly, included studies were largely based in Western countries and cultural differences in the examined associations should be considered. Thirdly, our review focused on clearly defined measures of both anxiety symptoms and eating disorder symptoms and excluded shared concepts such as ‘fear of weight gain’ or ‘appearance anxiety’. Given the natural overlap in symptoms and definitions of many psychological constructs, this may have introduced an artificial separation between anxiety symptoms and eating disorder symptoms.

## Conclusion

The current systematic review and meta-analysis have shown evidence for a bi-directional prospective relationship between anxiety symptoms and eating disorder symptoms in adolescence. However, the reviewed research has a range of limitations, which require attention in future research. Findings from this review have potential clinical implications in terms of prevention and early intervention for both anxiety symptoms and eating disorder symptoms.

## Supplementary Information

Below is the link to the electronic supplementary material.

Supplementary file1 (DOCX 35 KB)

Supplementary file2 (DOC 68 KB)

Supplementary file3 (DOCX 14 KB)

## Data Availability

The data and analysis code used for the meta-analyses is publicly available at https://osf.io/hf57n.
