# Supplementary material for: The prospective relationship between anxiety symptoms and eating disorder symptoms among adolescents: a systematic review and meta-analysis of a bi-directional relationship
Source: Eur Child Adolesc Psychiatry. 2024 Nov 7;34(6):1691–718. doi: 10.1007/s00787-024-02601-9 (PMC12198302; doi:10.1007/s00787-024-02601-9)
Supplement: Supplementary file 2 — Supplementary file2 (DOC 68 KB) [file 787_2024_2601_MOESM2_ESM.doc]

**Supplementary Figure 1.** Overlap of studies included in the meta-analyses


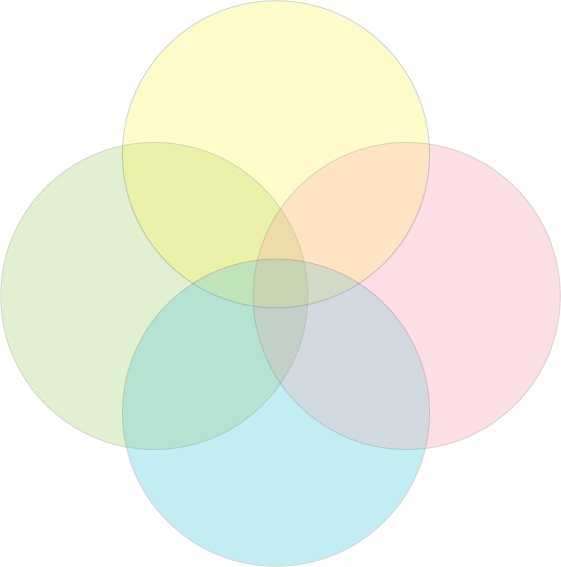


Johnson, 2002

Loose, 2023

Micali, 2015

Patton, 2008

Balantekin, 2017

Hautala, 2008

Herpertz-Dahlmann, 2015

Lieb,2016

Lloyd, 2019

Schaumberg, 2019

Sihvola, 2009

van Eeden, 2001

Benjet, 2016

Ranta, 2017

Robinson, 2020

Bardone-Cone, 2017

Hou, 2022

Levinson & Sala, 2016

Magson, 2023

McLaughlin, 2011

Minnich, 2014

Puccio, 2017

Bufferd, 2022

Hanback, 2009

Kidwell, 2017

Lee, 2019

Webb, 2021

*Note*: Green: Anxiety to ED, categorical; Blue: Anxiety to ED, continuous; Yellow: ED to anxiety, categorical; Pink: ED to anxiety, continuous
