# Supplementary material for: The prospective relationship between anxiety symptoms and eating disorder symptoms among adolescents: a systematic review and meta-analysis of a bi-directional relationship
Source: Eur Child Adolesc Psychiatry. 2024 Nov 7;34(6):1691–718. doi: 10.1007/s00787-024-02601-9 (PMC12198302; doi:10.1007/s00787-024-02601-9)
Supplement: Supplementary file 3 — Supplementary file3 (DOCX 14 KB) [file 787_2024_2601_MOESM3_ESM.docx]

Search terms by database

MEDLINE

(anxi* or fear or ruminat* or worry* or PTSD or OCD or generalized anxiety or phobi* or obsessi* or compulsi* or intrusive th* or shyness or nervousness) and (eating habits or disordered eating or eating disorder* or eating pathology or body image or body dissatisfaction or shape concerns or weight concerns or excessive exercise or diet* or restrictive eat* or dietary restraint or fast* or intention to lose weight or bulimi* or binge* or overeat* or night eat* or purg* or laxative or diuretic or vomit* or loss of control eat* or emotional eat*) and (teen* or youth* or adolescen* or juvenile* or young adult* or young person* or young people* or young m#n or young wom#n or high school* or child*). ab

PsychINFO

(anxi* or fear or ruminat* or worry* or PTSD or OCD or generalized anxiety or phobi* or obsessi* or compulsi* or intrusive th* or shyness or nervousness) and (eating habits or disordered eating or eating disorder* or eating pathology or body image or body dissatisfaction or shape concerns or weight concerns or excessive exercise or diet* or restrictive eat* or dietary restraint or fast* or intention to lose weight or bulimi* or binge* or overeat* or night eat* or purg* or laxative or diuretic or vomit* or loss of control eat* or emotional eat*) and (teen* or youth* or adolescen* or juvenile* or young adult* or young person* or young people* or young m#n or young wom#n or high school* or child*) ab,hw,id,ot,ti

SCOPUS

TITLE-ABS-KEY(("anxi*" OR {fear} OR {ruminat*} OR "worry*" OR {PTSD} OR {OCD} OR {generalized anxiety} OR {phobi*} OR {obsessi*} OR {compulsi*} OR {intrusive th*} OR {shyness} OR {nervousness}) AND ({eating habits} OR {disordered eating} OR {eating disorder*} OR "eating pathology" OR "body image" OR {body dissatisfaction} OR {shape concerns} OR {weight concerns} OR {excessive exercise} OR {diet*} OR {restrictive eat*} OR {fast*} OR {dietary restraint} OR {intention to lose weight} OR "bulimi*" OR {binge*} OR {overeat*} OR {night eat*} OR {purg*} OR {laxative} OR {diuretic} OR {vomit*} OR {loss of control eat*} OR {emotional eat*}) AND ( "teen*" OR "youth*" OR "adolescen*" OR "juvenile*" OR "young adult*" OR "young person*" OR {young people} OR {young m#n} OR {young wom#n} OR {high school*} OR "child*" ))

Web of science

((TS=(“anxi*” OR “fear” OR “ruminat*” OR “worry*” OR “PTSD” OR “OCD” OR “generalized anxiety” OR “phobi*” OR “obsessi*” OR “compulsi*” OR “intrusive th*” OR “shyness” OR “nervousness”)) AND TS=(“eating habits” OR “disordered eating” OR “eating disorder*” OR “eating pathology” OR “body image” OR “body dissatisfaction” OR “shape concerns” OR “weight concerns” OR “excessive exercise” OR “diet*” OR “restrictive eat*” OR “dietary restraint” OR “fast*” OR “intention to lose weight” OR “bulimi*” OR “binge*” OR “overeat*” OR “night eat*” OR “purg*” OR “laxative” OR “diuretic” OR “vomit*” OR “loss of control eat*” OR “emotional eat*”)) AND TS=(“teen*” OR “youth*” OR “adolescen*” OR “juvenile*” OR “young adult*” OR “young person*” OR “young people*” OR “young m#n” OR “young wom#n” OR “high school*” OR “child*”)

Embase

PsychArXiv

((“anxi*” OR “fear” OR “ruminat*” OR “worry*” OR “PTSD” OR “OCD” OR “generalized anxiety” OR “phobi*” OR “obsessi*” OR “compulsi*” OR “intrusive th*” OR “shyness” OR “nervousness”) AND (“eating habits” OR “disordered eating” OR “eating disorder*” OR “eating pathology” OR “body image” OR “body dissatisfaction” OR “shape concerns” OR “weight concerns” OR “excessive exercise” OR “diet*” OR “restrictive eat*” OR “dietary restraint” OR “fast*” OR “intention to lose weight” OR “bulimi*” OR “binge*” OR “overeat*” OR “night eat*” OR “purg*” OR “laxative” OR “diuretic” OR “vomit*” OR “loss of control eat*” OR “emotional eat*”) AND (“teen*” OR “youth*” OR “adolescen*” OR “juvenile*” OR “young adult*” OR “young person*” OR “young people*” OR “young m#n” OR “young wom#n” OR “high school*” OR “child*”))

ProQuest

summary(“anxi*” OR “fear” OR “ruminat*” OR “worry*” OR “PTSD” OR “OCD” OR “generalized anxiety” OR “phobi*” OR “obsessi*” OR “compulsi*” OR “intrusive th*” OR “shyness” OR “nervousness”) AND summary(“eating habits” OR “disordered eating” OR “eating disorder*” OR “eating pathology” OR “body image” OR “body dissatisfaction” OR “shape concerns” OR “weight concerns” OR “excessive exercise” OR “diet*” OR “restrictive eat*” OR “dietary restraint” OR “fast*” OR “intention to lose weight” OR “bulimi*” OR “binge*” OR “overeat*” OR “night eat*” OR “purg*” OR “laxative” OR “diuretic” OR “vomit*” OR “loss of control eat*” OR “emotional eat*”) AND summary(“teen*” OR “youth*” OR “adolescen*” OR “juvenile*” OR “young adult*” OR “young person*” OR “young people*” OR “young m#n” OR “young wom#n” OR “high school*” OR “child*”)
